# Inequities in COVID-19 Vaccination Coverage Among Pregnant Persons, by Disaggregated Race and Ethnicity — Massachusetts, May 2021–October 2022

**DOI:** 10.15585/mmwr.mm7239a2

**Published:** 2023-09-29

**Authors:** Hanna M. Shephard, Susan E. Manning, Eirini Nestoridi, Anne Marie Darling, Catherine M. Brown, Megan Hatch, Kathryn Ahnger-Pier, Sharon Pagnano, Darien Mather, Mahsa M. Yazdy

**Affiliations:** ^1^Bureau of Family Health and Nutrition, Massachusetts Department of Public Health; ^2^Division of Reproductive Health, National Center for Chronic Disease Prevention and Health Promotion, CDC; ^3^Bureau of Infectious Disease and Laboratory Sciences, Massachusetts Department of Public Health; ^4^Registry of Vital Records and Statistics, Massachusetts Department of Public Health.

**Summary**

**What is already known about this topic?**

Among pregnant persons in the United States, Hispanic or Latino (Hispanic) and non-Hispanic Black or African American persons experience the highest COVID-19 rates and the lowest COVID-19 vaccination coverage. Aggregation of race and ethnicity data can obscure within-group diversity and inequities.

**What is added by this report?**

Among 102,275 Massachusetts residents with pregnancies resulting in live birth during May 2021–October 2022, data disaggregation into 12 racial and 34 ethnic groups revealed inequities in COVID-19 vaccination coverage that were masked within all larger race and ethnicity groupings.

**What are the implications for public health practice?**

Disaggregating race and ethnicity data can uncover within-group differences in COVID-19 vaccination coverage that might guide tailored public health messaging.

## Abstract

National estimates suggest that COVID-19 vaccination coverage among pregnant persons is lower among those identifying as Hispanic or Latino (Hispanic) and non-Hispanic Black or African American. When examining COVID-19 vaccination coverage during pregnancy by race and ethnicity, however, data are typically limited to large, aggregate categories that might obscure within-group inequities. To address this, Massachusetts examined COVID-19 vaccination coverage among pregnant persons by combinations of 12 racial and 34 ethnic groupings. Among 102,275 persons with a live birth in Massachusetts during May 1, 2021–October 31, 2022, receipt of ≥1 dose of a COVID-19 vaccine before or during pregnancy was 41.6% overall and was highest among persons who identified as Asian (55.0%) and lowest among those who identified as Hispanic (26.7%). However, within all broad racial and ethnic groupings, disparities in COVID-19 vaccination coverage were identified when the data were disaggregated into more granular categories; for example, COVID-19 vaccination coverage ranged from 10.8%–61.1% among pregnant persons who identified as Hispanic. Disaggregated analyses reveal diverse experiences within broad racial and ethnic groupings. This information can be used to guide outreach to pregnant persons in communities with lower rates of COVID-19 vaccination coverage during pregnancy.

## Introduction

Despite mounting evidence that pregnancy is associated with elevated risk for severe COVID-19–associated illness and death (*1*–*3*), pregnant persons have lower COVID-19 vaccination coverage compared with nonpregnant persons of reproductive age (*1*). However, because COVID-19 vaccination can substantially reduce one’s risk for severe illness from COVID-19 (*4*), it is critical that all persons, including those who are pregnant or planning pregnancy, stay up to date with recommended COVID-19 vaccination. In addition, national data suggest that COVID-19 vaccination coverage is lower and rates of COVID-19 are higher among Hispanic or Latino (Hispanic) and non-Hispanic Black or African American (Black) pregnant persons (*1*). Vaccination access and outreach strategies are developed at state and local levels, yet only national-level estimates of COVID-19 vaccination coverage among pregnant persons are widely available. Moreover, data are often aggregated into six single race (American Indian or Alaska Native [AI/AN], Asian, Black, Native Hawaiian or other Pacific Islander [NH/OPI], and White) and ethnicity (Hispanic) groupings put forth by the Office of Management and Budget* and used by the U.S. Census Bureau,^†^ obscuring a diversity of within-group experiences and inequities (*4*). To examine COVID-19 vaccination coverage among pregnant persons in Massachusetts overall and to assess within-group inequities, a disaggregated, descriptive analysis of 12 racial and 34 ethnic groups was performed using COVID-19 vaccination data from the Massachusetts Immunization Information System (MIIS)^§^ linked to Massachusetts birth certificate data from the Registry of Vital Records and Statistics (RVRS).^¶^

## Methods

COVID-19 vaccination coverage was defined as the percentage of persons who had received ≥1 dose of a COVID-19 vaccine.** Coverage was estimated retrospectively among Massachusetts residents with a live birth during May 1, 2021–October 31, 2022, by deterministically linking COVID-19 vaccination data from MIIS with birth certificates, using various combinations of pregnant persons’ first and last name, date of birth, and street address. Because of potential missed linkages between MIIS and RVRS, mean imputation was used for possible matches to estimate an upper limit for COVID-19 vaccination coverage (*5*).

Given lower COVID-19 vaccination coverage and higher vaccine hesitancy rates experienced by persons who are pregnant or trying to become pregnant (*1*), coverage before^††^ or during pregnancy was examined separately from coverage after delivery.^§§^ COVID-19 vaccination was considered to have occurred during pregnancy if a person received any COVID-19 vaccine between the date of their last menstrual period (LMP) and their date of delivery. When LMP was missing, the newborn’s gestational age was used to ascertain the pregnancy window and whether vaccination occurred during this window.

Self-reported race and ethnicity were obtained from birth certificates of the pregnant person’s offspring; pregnant persons could select from a list^¶¶^^,^*** and write in all races, ethnicities, and tribes with which they identified. Persons were asked to first choose all ethnicities with which they identified followed by all races with which they identified. Self-reported race data were aggregated into the following nonmutually exclusive categories for analysis: AI/AN,^†††^ Asian,^§§§^ Black,^¶¶¶^ Hispanic,**** NH/OPI,^††††^ White,^§§§§^ and “another” race.^¶¶¶¶^ When disaggregating these racial categories, all other races and ethnicities with which a person identified were presented (e.g., a person identifying as both Black and Asian would be reflected in estimates for both groups). Thus, COVID-19 vaccination coverage was estimated overall, in these broad race groupings, and among more granular racial and ethnic subgroups and not limited to single-race categories. Race and ethnicity information was available for 98.6% and 98.5% of pregnant persons, respectively. Rates of COVID-19 vaccination coverage among racial and ethnic groups were not reported when the denominator was <10 or the rate of COVID-19 vaccination coverage multiplied by the number of persons in a group was 1–4. Rates of COVID-19 vaccination coverage and 95% CIs were calculated using SAS software (version 9.4; SAS Institute). This public health surveillance activity was reviewed by the Massachusetts Department of Public Health and CDC, deemed not research, and conducted consistent with applicable state and federal law and CDC policy.*****

## Results

**FIGURE 1:**
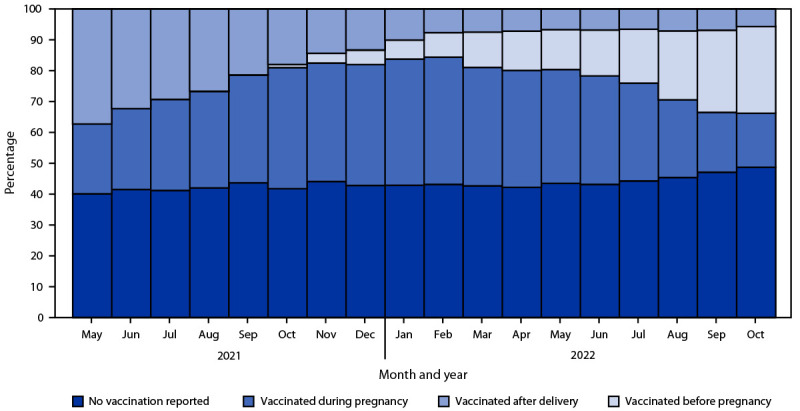
COVID-19 vaccination timing in relation to pregnancy as of October 31, 2022, by month of delivery — Massachusetts, May 1, 2021–October 31, 2022

Among 102,275 persons with a live birth occurring during May 1, 2021–October 31, 2022, COVID-19 vaccination coverage before or during pregnancy was 41.6% overall. COVID-19 vaccination coverage before or during pregnancy increased from May 2021 (22.6%) to April 2022 (50.6%), then declined slightly from May 2022 to October 2022 (45.7%) (Figure 1). The proportion of deliveries with no COVID-19 vaccination reported^†††††^ remained stable over time (mean = 43.4%; range = 40.1%–48.7%). COVID-19 vaccination coverage, examined irrespective of the specific pregnancy window (i.e., vaccination occurring before, during, or after pregnancy), was 56.7%. However, when the mean COVID-19 vaccination coverage among possible MIIS-RVRS matches (N = 19,858) was imputed, coverage was estimated to be as high as 73.1%.

**FIGURE 2:**
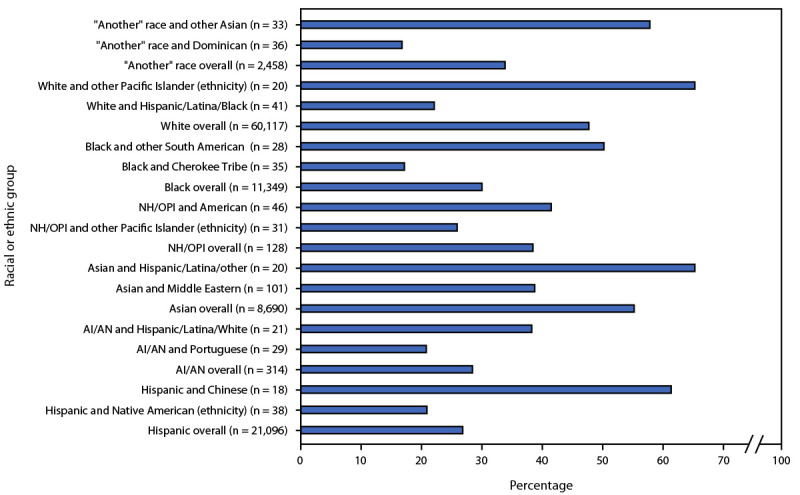
COVID-19 vaccination coverage* before or during pregnancy by race and ethnicity (large groupings overall and racial and ethnic subgroups with highest and lowest rates of coverage within these large groupings)^†^^,^^§^^,^^¶^^,^**^,^^††^^,^^§§^^,^^¶¶^^,^***^,^^†††^ among pregnancies resulting in live birth — Massachusetts, May 1, 2021–October 31, 2022 **Abbreviations:** AI/AN = American Indian or Alaska Native; NH/OPI = Native Hawaiian or other Pacific Islander. * Receipt of >1 dose of a COVID-19 vaccine (primary series or any subsequent dose). ^†^ The Hispanic overarching category includes persons who selected one or more of the following races on the birth certificate: Hispanic/Latina/Black, Hispanic/Latina/White, or Hispanic/Latina/other. ^§^ Massachusetts birth certificates include an AI/AN option for race and a Native American option for ethnicity. Whereas the AI/AN overarching category includes those who identify as being racially AI/AN, there are also persons who identify as ethnically Native American (and who might identify as racially AI/AN). ^¶^ The AI/AN overarching category includes persons who selected AI/AN as their race (or one of their races) on the birth certificate. ** The Asian overarching category includes persons who selected Asian as their race (or one of their races) on the birth certificate. ^††^ The NH/OPI overarching category reflects persons who selected one or more of the following races on the birth certificate: Guamanian or Chamorro, Native Hawaiian, Samoan, or other Pacific Islander. ^§§^ The Black or African American (Black) overarching category includes persons who selected Black as their race (or one of their races) on the birth certificate. ^¶¶^ Among Black pregnant persons, the lowest rate of COVID-19 vaccination uptake before or during pregnancy was tied between three groupings: persons who also identified as other South American ethnicity (50.0%), Mexican ethnicity (50.0%), or Asian Indian ethnicity (50.0%). *** The White overarching category includes persons who selected White as their race (or one of their races) on the birth certificate. ^†††^ The “another” race category includes persons who selected “other race not listed” on the birth certificate as one of their races or for whom no race category was selected or who opted not to identify their race, so the birth registrar indicated this as a refusal.

Coverage before or during pregnancy was highest among persons who identified as Asian (55.0% overall; subgroup range = 38.6%–65.0%) and lowest among Hispanic persons (26.7% overall; subgroup range = 20.8%–61.1%) (Figure 2). Overall, race and ethnicity–specific COVID-19 vaccination coverage before or during pregnancy was 28.3% among AI/AN pregnant persons (subgroup range = 20.7%–38.1%); 29.9% among Black pregnant persons (subgroup range = 17.1%–50.0%); 38.3% among NH/OPI pregnant persons (subgroup range = 25.8%–41.3%); and 47.5% among White pregnant persons (subgroup range = 22.0%–65.0%) (Supplementary Table, https://stacks.cdc.gov/view/cdc/133102).^§§§§§^ Substantial variation in coverage was also observed among those who identified with another race (33.7%; subgroup range = 16.7%–57.6%).

## Discussion

In Massachusetts, COVID-19 vaccination coverage before or during pregnancy was lowest among persons who identified as Hispanic and highest among those who identified as Asian. These findings are consistent with those of a previous study of COVID-19 vaccination during pregnancy that reported higher rates of COVID-19 vaccination among Asian and White pregnant persons compared with Black and Hispanic pregnant persons (*1*). However, the present study identified wide heterogeneity within all racial groups that was masked in aggregate results (e.g., COVID-19 vaccination coverage was lower among those who identified as Asian and Laotian [39.7%] compared to those who identified as Black and Asian Indian [50.0%]). A community-informed analysis of COVID-19 related deaths in Hawaii demonstrated similar heterogeneity among Native Hawaiian, Pacific Islander, and Asian subpopulations, and highlighted the importance of disaggregating state-level data to identify inequities (*6*). That study also emphasized the importance of highlighting public health concerns in certain communities without further stigmatizing institutionally underserved groups (*6*). The current analysis demonstrates that disaggregation of data allows for a more in-depth examination that might reveal inequities. These findings suggest that disaggregation of race and ethnicity data at the local level can be used to develop public health action tailored to individual communities. This action could include developing culturally relevant messages and materials translated into the preferred languages of communities with lower rates of COVID-19 vaccination coverage and engaging trusted messengers to address vaccine hesitancy.^¶¶¶¶¶^

Race and ethnicity categorizations are socially constructed and not based on biologic differences. However, racial and ethnic health inequities persist, reflecting, in part, structural and institutional racism,****** which drives barriers to health care access, medical mistrust, and marginalization among persons from some racial and ethnic groups (*7*). The observed inequities in COVID-19 vaccination coverage among AI/AN, Black, and Hispanic pregnant persons in Massachusetts might be considered within the context of racism as a root cause. Persons who are marginalized on the basis of systemic inequalities stemming from racism are disproportionately affected by COVID-19, and the pandemic could exacerbate existing inequities in maternal morbidity and mortality (*8*). 

### Limitations

The findings in this report are subject to at least eight limitations. First, COVID-19 vaccination coverage was estimated by linking reports of COVID-19 vaccination to birth certificates for completed pregnancies resulting in a live birth; therefore, vaccination coverage among persons who experienced early pregnancy losses, terminations, or stillbirths were not reflected in the analysis. Second, the deterministic linkage process relied on exact matching between linking variables in MIIS and RVRS, likely underestimating COVID-19 vaccination coverage overall. Moreover, deterministic linkages have been found to be less accurate for non-English names, resulting in potential differences in linkage completeness by racial and ethnic subgroup and further underestimation of coverage among subgroups with higher proportions of non-English names (*9*). Third, not all COVID-19 vaccinations are represented in MIIS (e.g., vaccinations occurring out of state or at a federal agency not required to report to MIIS), which might result in underestimation of vaccination coverage in the present analysis. To address this, an upper limit was estimated for COVID-19 vaccination coverage using mean imputation. Fourth, if missed linkages between MIIS and RVRS did not occur at random, mean imputation would result in a biased estimate. Fifth, categories for race and ethnicity overlap.^††††††^^,^^§§§§§§^ Sixth, small numbers for some racial and ethnic subgroups resulted in unstable estimates with wide CIs. Seventh, Massachusetts data might not be generalizable to other jurisdictions. Finally, the use of non-mutually exclusive race and ethnicity categories limits the ability to compare groupings; however, reflecting persons who hold multiple racial and ethnic identities in all the groups with which they identified was critical to respect self-identification.

### Implications for Public Health Practice

Disaggregated analyses reveal diverse experiences within broad racial and ethnic groupings. Similar analyses can be used at a state or local level to guide outreach to pregnant persons in communities with lower rates of COVID-19 vaccination coverage during pregnancy. Moreover, identifying and centering racial and ethnic subgroups and communities with lower rates of vaccination coverage in COVID-19 prevention and mitigation strategies, such as developing tailored health education materials for and increasing COVID-19 vaccination outreach in these communities, could more effectively address racial and ethnic health inequities.

## Footnotes

*
https://www.govinfo.gov/content/pkg/FR-1997-10-30/pdf/97-28653.pdf

†
https://www.census.gov/

§
https://www.mass.gov/massachusetts-immunization-information-system-miis

¶
https://www.mass.gov/vital-records-data-and-publications

** COVID-19 vaccine dose includes primary series vaccinations and any subsequent dose.

†† Receipt of ≥1 dose of any COVID-19 vaccine (primary series or any subsequent dose) before the beginning of the pregnancy.

§§ Receipt of ≥1 dose of any COVID-19 vaccine (primary series or any subsequent dose) after delivery.

¶¶ Massachusetts birth certificates include the following race categories: American Indian/Alaska Native, Asian, Black, Guamanian or Chamorro, Hispanic/Latina/Black, Hispanic/Latina/White, Hispanic/Latina/other, Native Hawaiian, Samoan, White, other Pacific Islander, other race not listed; for ethnicity: African, African-American, American, Asian Indian, Brazilian, Cambodian, Cape Verdean, Caribbean Islander, Chinese, Colombian, Cuban, Dominican, European, Filipino, Guatemalan, Haitian, Honduran, Japanese, Korean, Laotian, Mexican/Mexican American/Chicano, Middle Eastern, Native American, (specify tribal nation or nations), Portuguese, Puerto Rican, Russian, Salvadoran, Vietnamese, other Asian, other Central American, other Pacific Islander, other Portuguese, other South American, and other ethnicity not listed.

***
https://www.mass.gov/info-details/resources-for-preparing-vital-records

††† The AI/AN overarching category includes persons who selected AI/AN as their race (or one of their races) on the birth certificate.

§§§ The Asian overarching category includes persons who selected Asian as their race (or one of their races) on the birth certificate.

¶¶¶ The Black overarching category includes persons who selected Black as their race (or one of their races) on the birth certificate.

**** The Hispanic overarching category includes persons who selected one or more of the following categories on the birth certificate as their race: Hispanic/Latina/Black, Hispanic/Latina/White, or Hispanic/Latina/other.

†††† The NH/OPI overarching category reflects persons who selected one or more of the following races on the birth certificate: Guamanian or Chamorro, Native Hawaiian, Samoan, or other Pacific Islander.

§§§§ The White overarching category includes persons who selected White as their race (or one of their races) on the birth certificate.

¶¶¶¶ The “another” race category includes persons who selected “other race not listed” on the birth certificate as one of their races or for whom no race category was selected or who opted not to identify their race, so the birth registrar indicated this as a refusal.

***** 45 C.F.R. part 46, 21 C.F.R. part 56; 42 U.S.C. Sect. 241(d); 5 U.S.C. Sect. 552a; 44 U.S.C. Sect. 3501 et seq.

††††† No COVID-19 vaccination was reported for these persons in MIIS. This could be because vaccination occurred out of state or at a federal agency not required to report into MIIS (e.g., Indian Health Service), a missed linkage between MIIS and vital records because of discrepancies in linking variables (e.g., name and address), or because this person did not receive any COVID-19 vaccines.

§§§§§ To maintain confidentiality, rates of COVID-19 vaccination coverage among racial and ethnic groups were not reported when the denominator was <10 or the numerator would allow for the calculation of any other cells with values 1–4.

¶¶¶¶¶
https://www.cdc.gov/vaccines/covid-19/hcp/tailoring-information.html

******
https://www.cdc.gov/media/releases/2021/s0408-racism-health.html

†††††† Massachusetts birth certificates include other Pacific Islander as both race and ethnicity categories. Whereas the NH/OPI overarching category includes those who identify as being racially other Pacific Islander, there are also persons who identify as ethnically other Pacific Islander (and who might identify as racially other Pacific Islander).

§§§§§§ Massachusetts birth certificates include an AI/AN option for race and a Native American option for ethnicity. Whereas the AI/AN overarching category includes those who identify as being racially AI/AN, there are also persons who identify as ethnically Native American (and who might or might not identify as racially AI/AN).
